# Unraveling the Population Structure of *Temnocephala iheringi* Across Host Associations and Geographic Regions

**DOI:** 10.3390/biology15131020

**Published:** 2026-06-26

**Authors:** Agustina Zivano, Carolina Noreña, Samantha A. Seixas, Francisco Brusa, Cristina Damborenea

**Affiliations:** 1 División Zoología Invertebrados, Museo de La Plata (FCNyM-UNLP), La Plata 1900, Argentina; fbrusa@fcnym.unlp.edu.ar; 2 Departamento de Biodiversidad y Biología Evolutiva, Museo Nacional de Ciencias Naturales (MNCN–CSIC), 28006 Madrid, Spain; norena@mncn.csic.es; 3 Independent Researcher, Porto Alegre 91210-001, RS, Brazil; seixas.sa@gmail.com; 4 Consejo Nacional de Investigaciones Científicas y Técnicas (CONICET), La Plata 1900, Argentina

**Keywords:** ectosymbiont, Ampullariidae, Temnocephalidae, COI, South America

## Abstract

Commensalism, a frequent type of interaction among freshwater invertebrates, remains poorly studied. Some turbellarians (Platyhelminthes: Temnocephalidae) are specialized obligate commensals of crustaceans, mollusks, insects, and turtles. In the Neotropics, *Temnocephala iheringi* inhabits the mantle cavity of snails (Mollusca: Gastropoda) from Pantanal (Brazil) to the Pampean region of Argentina, where several species serve as hosts. This study aimed to molecularly characterize several populations of *T. iheringi* and to analyze their genetic and morphological variability across different host species and geographic areas. Using the mitochondrial COI marker, we assessed populations associated with five of its seven known host species through phylogenetic reconstructions, species delimitation approaches, and haplotype network analyses. Combined with morphological data, results support COI as an effective identification tool for Temnocephalidae. Several genetic lineages were identified and were largely congruent with collection localities. However, specimens associated with hosts displaying high dispersal capabilities (i.e., *Pomacea canaliculata* and *P. maculata*) showed low mitochondrial genetic differentiation and minimal phylogenetic structure across large distances, which may be consistent with recent dispersal and/or ongoing connectivity among populations. These findings provide new insights into the evolutionary dynamics of this specific temnocephalid–snail association. Given that some hosts are highly invasive and even considered pests in several countries, the data and genetic sequences generated in this study may prove valuable for future research on symbiont diversity and dispersal.

## Simple Summary

Despite being common in nature, commensalism—a relationship where one organism benefits from another without causing it harm—is poorly studied in freshwater environments. This study focused on a tiny flatworm, *Temnocephala iheringi*, which lives exclusively associated with South American freshwater snails. This study aimed to map the genetic and anatomical differences between these worms across various regions and snail species. By analyzing a specific DNA “barcode” (the COI marker), we discovered that while many worm populations are genetically unique to their local areas, those living on species with high-dispersal capabilities are very similar over vast distances. These findings provide new insights into the evolutionary dynamics of this specific commensal–snail association.

## 1. Introduction

Temnocephalids (Platyhelminthes, Rhabdocoela, Temnocephalidae) are obligate commensals primarily associated with freshwater crustaceans [1]. In the Neotropical region, the endemic genus *Temnocephala* Blanchard, 1849, is widely distributed. In addition to their association with crustaceans, several species in this region are obligate commensals of mollusks, insects, and turtles [2]. Each species is typically restricted to a closely related group of hosts and occupies a specific micro-habitat, suggesting a robust ecological and evolutionary relationship between the commensal and its host.

In the Neotropics, most studies on *Temnocephala* spp. have been morphological, focusing on species identification and host records. To date, identification has relied on anatomy, specifically the morphology of the genital apparatus and syncytial excretory plates, with the stylet being the most relevant diagnostic feature [3,4]. Only a few investigations have adopted an integrative approach by considering host interactions [5,6,7,8]. Furthermore, there is a notable absence of molecular studies addressing species identification and population dynamics.

Among the nearly 40 species of *Temnocephala*, *T. iheringi* Haswell, 1893, is both widely distributed and extensively studied. It is associated with freshwater snails of the family Ampullariidae (Caenogastropoda), ranging from central Brazil to Uruguay and the Southern Pampean region in Argentina [9,10,11,12]. The host species most frequently cited in the literature is *Pomacea canaliculata* (Lamarck, 1822), although frequent misidentifications occur, often confusing it with *Pomacea maculata* G. Perry, 1810 [13], which also hosts *T. iheringi*. Beyond these two ampullariids, other hosts include *P. megastoma* (G. B. Sowerby I, 1825), *Asolene platae* (Maton, 1811), *Pomacea linnaei* (R. A. Philippi, 1852), and *Marisa planogyra* Pilsbry, 1933. Recently, *T. iheringi* was also found in association with *Pomacea americanista* (Ihering, 1919) and *Chilina iguazuensis* Gutiérrez Gregoric & Rumi, 2008 (Heterobranchia, Chilinidae) [12].

The obligate and specialized interaction between *Temnocephala iheringi* and its hosts involves ecological patterns and evolutionary processes that remain poorly understood. Most recent studies have focused primarily on morphological variability within species [11,12], revealing considerable intraspecific variation and attempting to elucidate whether such variability is associated with host species or geographic distribution, without taking into account genetic differentiation. Therefore, investigating these patterns with an integrative approach is of significant biological and evolutionary relevance.

To address these knowledge gaps, we molecularly characterized several populations of *T. iheringi* and evaluated their morphological and genetic variability in relation to host species and geographic distribution. Using the mitochondrial COI marker, we assessed population-level variability in specimens of *T. iheringi* associated with five of its seven known host species. By integrating morphological and molecular data, this study provides the first molecular assessment of a Neotropical temnocephalan and offers new insights into the diversification patterns of this host-associated taxon.

## 2. Materials and Methods

### 2.1. Biological Sampling

**Figure 1:**
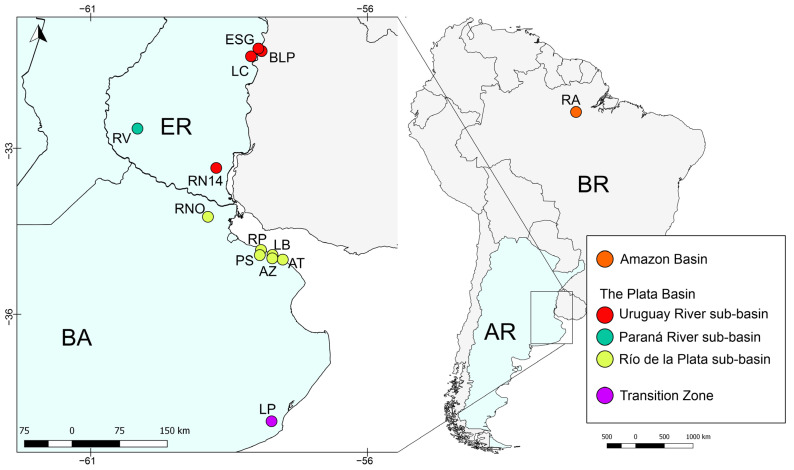
Map of the sampling sites by hydrographic basin.

Sampling was carried out on 12 sites in Argentina and one in Brazil (Table 1, Figure 1). Five host species were collected: *P. linnaei*, *P. canaliculata*, *P. maculata*, *P. megastoma*, *Asolene platae*. Additionally, temnocephalans from the shrimp *Palaemon argentinus* (Nobili, 1901) were sequenced as outgroup (from an artificial pond in Berisso, Buenos Aires, Argentina, −34.887778, −57.828333, 10 March 2015, 14 April 2015, 18 August 2015).

**Figure 2:**
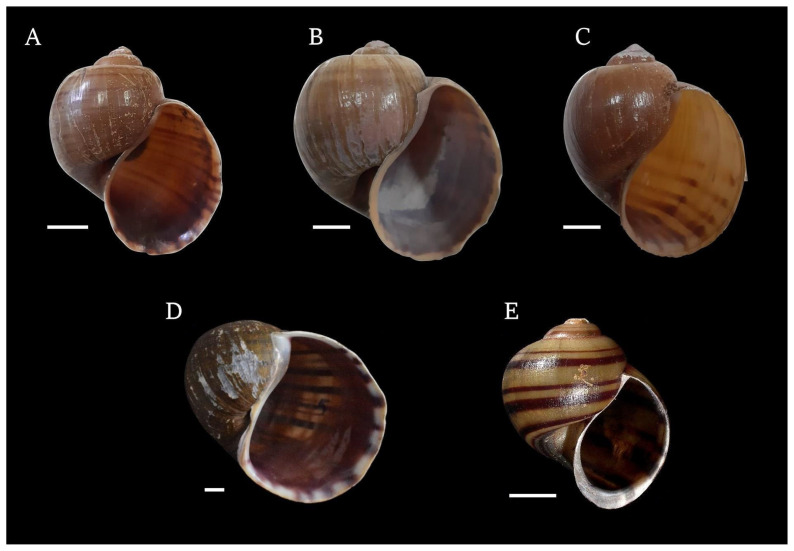
Host species. (**A**). *Pomacea canaliculata*; (**B**). *Pomacea maculata*; (**C**). *Pomacea linnaei*; (**D**). *Pomacea megastoma*; (**E**). *Asolene platae*. Scale bar 1 cm.

Hosts were found in both lentic and lotic environments (among the vegetation or on the mud, sand, or rocks). They were collected by hand or with a hand net, put in individual plastic jars with water from the site, and transported alive to the field laboratory or to the Museo de La Plata. Once at the laboratory, hosts were relaxed with menthol to facilitate the release of symbionts from the mantle cavity. The hosts were later dissected to examine the mantle cavity in search of any temnocephalans that did not leave the snails during relaxation. Host identification followed de Castellanos & Fernández [14] and Cowie & Thiengo [15] (Figure 2), keeping up with current nomenclature on MolluscaBase [16]. To distinguish *P. maculata* from *P. canaliculata*, we followed Hayes et al. [13], basing the distinction on the angulation of the whorl shoulder and a distinct pigmentation of the inner pallial lip in *P. maculata*.

Temnocephalans were fixed in 100° ethanol. After fixation, temnocephalans were sectioned at the level of the posterior limit of the intestine. For morphological species identification, the posterior half of the animals was cleared with polyvinyl-lactophenol to observe the penial stylet, which is a diagnostic feature [3,4,17,18]. The anterior half of the specimens were used for DNA extraction. This method ensures that each sequence has a voucher with the morphological characteristics [1]. In some cases, due to small size, the whole specimen was used for DNA extraction, so those sequences do not have morphological voucher. Extra temnocephalans were fully clarified. Specimen vouchers were deposited in the Helminthological Collection of the Museo de la Plata.

### 2.2. Morphological Identification

**Figure 3:**
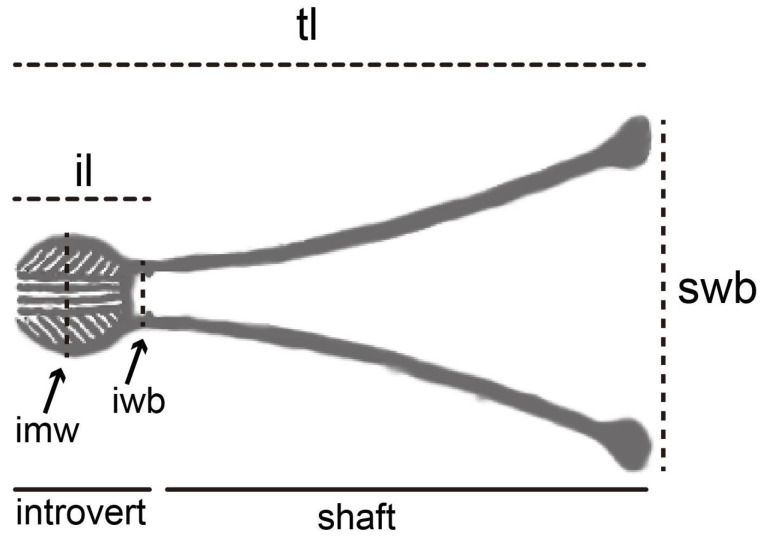
Diagram of a penial stylet of *Temnocephala* sp. showing its parts and the measurements taken. il, introvert length; imw, introvert maximum width; iwb, introvert width at base; swb, shaft width at base; tl, total length.

Temnocephalans were identified based on the morphology of the penial stylet. Penial stylets from adult *T. iheringi* specimens were measured in µm (stylet total length, shaft width at base, introvert length, introvert width at base, maximum introvert width at level of swelling) with a graduated ocular piece and photographed with a Zeiss Axiocam 208 (Carl Zeiss GmbH, Göttingen, Germany)color camera attached to a Zeiss Axiostar plus microscope, following Seixas et al. [10] (Figure 3). For some specimens, only measurements from the stylet introvert could be taken, given that the shafts were broken or folded during preparation. Also, in those cases where the whole specimen was used for DNA extraction, no measurements could be taken. The temnocephalans associated with *P. argentinus* were identified as *Temnocephala digitata* Monticelli, 1902, by morphological characteristics of the penial stylet, according to Zivano et al. [8].

### 2.3. DNA Extraction, Amplification, and Sequencing

DNA was extracted with the Wizard^®^ Genomic DNA Purification Kit (Promega Corp., Madison, WI, USA). A fragment of the COI gene was amplified by PCR with concentrations as follows: 0.1 mM dNTPs, 0.2 mM primers, 1× PCR buffer, and 1–2 U Taq polymerase (GoTaq^®^, Promega Corp., Madison, WI, USA), in a final volume of 25 µL. The primers used were “425F” (5′-GGNGCTAGNTCNATWTTAGGRGC-3′) and “new 1200R” (5′-CCATTGAWAMNACATAATGAAAATG-3′) [19]. The thermal cycle included a 5′ denaturalization period at 94 °C, followed by 40 cycles of 45″ at 94 °C, 45″ at 54 °C, and 1′ at 72 °C, with a final extension period of 7′ at 72 °C. After electrophoresis gel verification, the PCR products were sent to Macrogen Inc. (Seoul, Republic of Korea) for purification and double-strand sequencing. Some of the laboratory work was carried out at the Museo de Ciencias Naturales of Madrid. In this case, the PCR products were purified with ExoSAP-IT^®^ (Thermo Fisher Scientific Inc., Waltham, MA, USA) and sent for sequencing (forward strand only) to Secugen S.L. (Madrid, Spain).

The obtained sequences were edited with Chromas 2.6.6 (https://technelysium.com.au, accessed on 2 November 2022), and contigs were assembled on BioEdit 7.0.4.1 [20]. BLAST+ 2.13.0 was used to verify the sequences against the GenBank database (https://www.ncbi.nlm.nih.gov/genbank/, accessed on 3 November 2022). For the bioinformatic analyses, a sequence of the Australian species *Temnosewellia fax* Sewell, Cannon & Blair, 2006, downloaded from GenBank (KX095350.1), and the three newly obtained sequences from *Temnocephala digitata* were used to root the phylogenetic trees.

Sequences were aligned on MAFFT 7 with default parameters [21,22]. MEGA X10.1.6 [23] was used to check the reading frame of the alignment by translating it from nucleotides to amino acids following the Flatworms Genetic Code. Uncorrected paired distances (*p*-distance) were calculated on MEGA X. Mean distances within and between sampling sites and within and between temnocephalans from different host species, as well as within and among lineages, were also calculated, with 10,000 bootstrap replicates.

### 2.4. Phylogenetic Relationship and Species Delimitation

For phylogenetic analyses, the dataset was partitioned by codon position to allow for different substitution models. For Maximum Likelihood (ML), we used IQ-Tree 1.6.12 [24,25]. The nucleotide substitution models, selected with the integrated tool ModelFinder [26], were TIM + F + G4 for the first codon position, F81 + F + I for the second codon position, and TIM2 + F + G4 for the third codon position. Node support was calculated with ten thousand Ultrafast Bootstrap, and SH-aLRT replicates [27,28,29]. Bayesian Inference (BI) was run on Mr. Bayes 3.2.2 [30], with the following models selected by Partition Finder [29,31]: GTR + G, F81 + I, and GTR + G for each codon position, respectively. The analysis was run for 20 million generations with a 25% burn-in.

For species delimitation, ASAP–based on the alignment matrix—[32] and mPTP–based on the obtained trees—(https://mptp.h-its.org/#/tree, accessed on 9 April 2023; [33]) were used, both with default parameters.

### 2.5. Genetic Diversity Analyses

Haplotype diversity for *T. iheringi* was calculated with DnaSP v6 [34]. Haplotype networks were made with PopART 1.7 using the TCS method [35].

### 2.6. Geographic Distance and Morphometric Analyses

To check for correlation between genetic and geographic distances, a Mantel test [36] was run on R v4.0.3 (https://www.R-project.org/, accessed on 13 November 2020) using the package “vegan” [37], with 1000 permutations of Pearson’s correlation coefficient. Matrices used were pairwise total genetic distances and geographic distances measured overland (Supplementary Tables S2 and S3).

A multivariate analysis was used to investigate whether the observed stylet morphological variation is associated with host species, geographic distribution, or a combination of both factors. A principal coordinates analysis (PCoA) was run on Past 3.25 [38] with Gower’s index. Only adult specimens were included in the analysis.

## 3. Results

The average abundance of temnocephalans varied among the sampled locations (Table 2). The highest mean abundance was found at Las Palmeritas Beach, with 34 temnocephalans per host (*P. megastoma*), and the lowest was recorded at Riacho Victoria with only 0.60 commensals per host (*A. platae*).

Morphological identification based on the penial stylet allowed us to identify 59 specimens of *T. iheringi* and two of *Temnocephala* sp., from which COI sequences were successfully amplified. Also, three sequences of *T. digitata* were obtained (outgroup). Microphotographs of the penial stylet of the *T. iheringi* vouchers are provided (Supplementary Figure S1). GenBank accession numbers and museum deposit numbers, with the corresponding localities and hosts of the analyzed specimens, are listed in Table 2.

### 3.1. Genetic Distances

The genetic alignment had a length of 648 bp (Supplementary Table S1). Pairwise distances indicated divergences of 15.42% between *Temnocephala iheringi* and *Temnocephala* sp., 17.54% between *T. digitata* and *Temnocephala* sp., and 18.45% between *T. iheringi* and *T. digitata*. *T. iheringi* showed high internal variability, with a mean intraspecific distance of 6.38%.

Considering host association, specimens from *Pomacea canaliculata* and *P. maculata* were the closest (1.91%), while the highest distance was found between *P. megastoma* and *P. maculata* (12.60%). Regarding mean distances within hosts, *P. canaliculata* showed the highest variability (2.98%), while *P. linnaei* and *A. platae* showed the lowest (0.40 and 0.41%, respectively).

Comparatively, considering genetic distances between sampling sites, BLP and PS were the most distant (12.69%), while AZ and PS were the most similar (0.13%). Mean distances within sites showed the highest variability in RN14 (3.22%), while PS and BLP showed no variability (0%). All genetic distances are provided in Supplementary Table S2.

### 3.2. Phylogenetic Analyses and Species Delimitation

**Figure 4:**
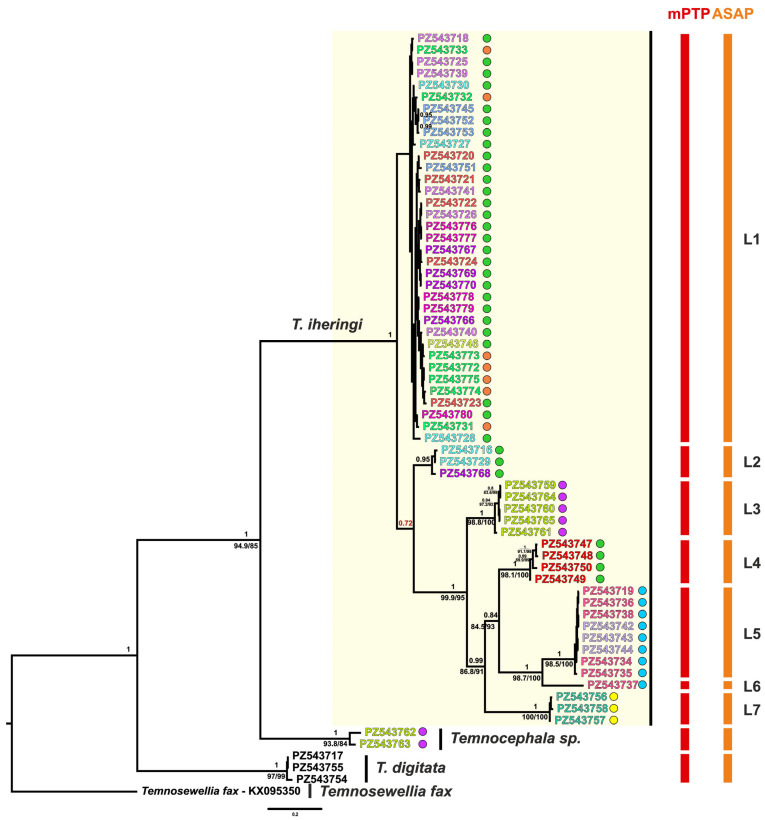
Bayesian Inference tree colored by locality. Posterior probability (over branches) and Sh-aLRT/Ultrafast Bootstrap (below branches, ML tree—Supplementary Figure S2) values are shown. Lineages obtained by species delimitation methods are shown with red (mPTP) and orange bars (ASAP). Colored dots indicate host species (light blue: *Pomacea megastoma*; green: *Pomacea canaliculata*; yellow: *Asolene platae*; orange: *Pomacea maculata*; purple: *Pomacea linnaei*). Outgroup sequences associated with crustacean hosts are in black.

Phylogenetic analyses supported the monophyly of *T. iheringi* and also of *T. digitata*. Two of the specimens from Brazil formed a distinct clade, sister to *T. iheringi*. Within *T. iheringi*, the phylogenetic structure revealed a large basal clade representing an evolutionary lineage with low internal variability, which includes almost all specimens from Buenos Aires province (AZ, AT, LB, RNO, PS, RP, LP), and three from Entre Ríos province (RN14), and was associated with *P. canaliculata* and *P. maculata*. Most of the remaining specimens studied formed clades by host species and localities: BLP, ESG, RV, LC (from Entre Ríos province), and RA (from Brazil). The first two are associated with *P. megastoma*; RV with *A. platae*; LC with *P. canaliculata*; and RA with *P. linnaei*. A small group formed by the remaining two specimens from R14 and one from Laguna de los Padres (Buenos Aires Province) showed unstable phylogenetic placement: as sister to the locality-structured clades in the BI tree (Figure 4, *pp* = 0.72, collapsed) and at the base of the *T. iheringi* clade in the ML tree (see Supplementary Figure S2).

Species delimitation by ASAP and mPTP yielded congruent results. Both methods separated *T. iheringi* from *T. digitata* and *Temnocephala* sp., and identified seven lineages within *T. iheringi*.

Specimens associated with *Pomacea linnaei*, *P. canaliculata*, and *Asolene platae* from Brazil and Entre Ríos Province in Argentina formed three distinct lineages (L3, L4, and L7, respectively). Specimens collected from *P. megastoma* in Entre Ríos Province grouped within another lineage (L5), although one specimen from Salto Grande Reservoir (ESG) in Entre Ríos Province constituted a separate lineage (L6). Most specimens from Buenos Aires Province, together with three specimens from RN14 in Entre Ríos, formed the largest lineage (L1), associated with *P. canaliculata* and *P. maculata*. In contrast, the remaining specimens from *P. canaliculata* were grouped in lineage L2, including one specimen from Laguna de los Padres in Buenos Aires Province and two from RN 14 in Entre Ríos.

### 3.3. Haplotype Diversity

**Figure 5:**
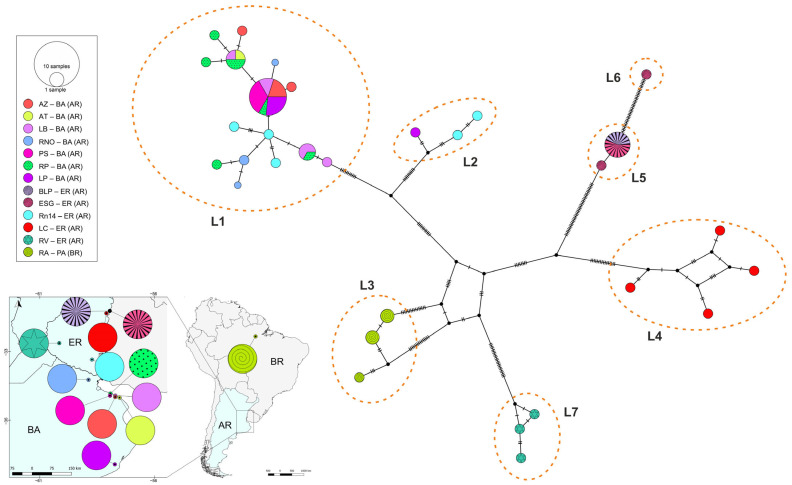
Haplotype network for *Temnocephala iheringi*. Colors indicate localities, and circle size indicates how many specimens share each haplotype. Host species are indicated by filling pattern: dotted, *P. maculata*; flat, *P. canaliculata*; spiraled, *P. linnaei*; starred, *A. platae*; striped, *P. megastoma*. Groups are numbered by lineage.

Among the 59 sequences for *T. iheringi*, 31 haplotypes were identified (Haplotype diversity (Hd): 0.92), 24 of which were unique (Table 3). Haplotype networks recovered the same seven lineages identified in phylogenetic and species delimitation analyses, separated by at least 25 nucleotide substitutions (Figure 5). Within lineages, haplotypes differed by 1–13 substitutions. The most common haplotype was shared by 15 specimens from different localities in Buenos Aires Province (AZ, LB, RP, PS, and LP) and two host species: *P. canaliculata* and *P. maculata* (L1).

Two localities (BLP and PS) showed only shared haplotypes, while three sampling localities from Entre Ríos Province (RV, RN14, and LC), each a distinct lineage, showed only unique haplotypes. The ESG from Entre Ríos Province showed both common and unique haplotypes, one of them separated into a lineage of its own (L6) with at least 39 substitutions.

Four out of five specimens from LP (Buenos Aires Province) shared the most common haplotype, while the remaining specimens showed a unique haplotype and grouped within lineage L2, along with two temnocephalans from RN14 (Entre Ríos). The specimens from Brazil (RA) did not share haplotypes with any of the Argentinean specimens.

### 3.4. Geographic Distance and Morphometric Analyses

Mantel’s test between genetic and geographic distances among populations revealed no significant evidence of correlation (*r* = 0.2134, *p* = 0.31).

**Figure 6:**
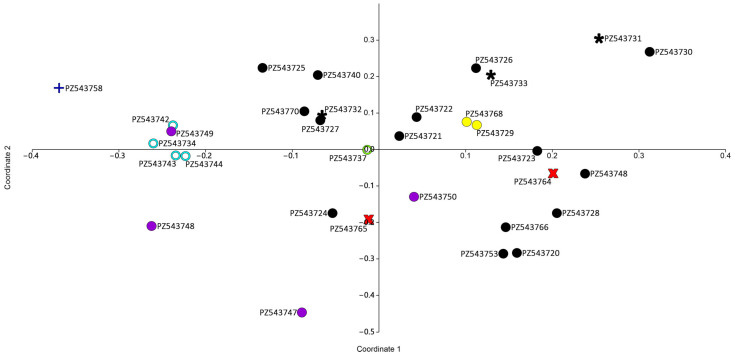
Principal coordinates analysis for penial stylet measurements from *Temnocephala iheringi* specimens. Color indicates lineages: black, L1; blue, L7; green, L6; light blue, L5; purple, L4; red, L3; yellow, L2. Shape indicates host species: **+** = *Asolene platae*; **×** = *Pomacea linnaei*; **•** = *Pomacea canaliculata*; * = *Pomacea maculata*; **○** = *Pomacea megastoma*.

Principal coordinates analysis (PCoA) based on measurements of the penial stylet of *T. iheringi* (Appendix A, Table A1) did not separate specimens according to host species or locality (Figure 6). Although some clustering was observed for specimens from Balneario Las Palmeritas (BLP) and Embalse Salto Grande (ESG), and in specimens from L2, these were not clearly distinct from others. The lineages identified by species delimitation (indicated by colors in Figure 6) were largely dispersed and overlapping, with the most evident grouping again corresponding to BLP, ESG, and L2.

## 4. Discussion

The species of *Temnocephala* are traditionally identified based on morphological characters of the penial stylet, other features of the reproductive system, and the number and morphology of the syncytial plates in the epidermis. To date, no studies in the Neotropical region have used molecular markers to confirm morphological identifications, and the genetic information currently available for Temnocephalidae in public databases is strongly biased toward species from Australia, New Zealand, Southeast Asia, and Europe [1] (and references therein). Consequently, Neotropical species remain markedly underrepresented.

In the present study, we sequenced a region of the mitochondrial gene COI from 59 specimens of *Temnocephala iheringi* from five host species and from different localities of Argentina and Brazil, expanding its distribution range 1500 km north of its northernmost record (Ypiranga Farm, Poconé, Mato Grosso, Brazil [11]), and into a new biome (Amazonia). Molecular identification based on phylogenetic inference was congruent with traditional morphological methods, confirming the monophyly of *T. iheringi* and *T. digitata*. Although species delimitation analyses recovered seven groups within *T. iheringi*, evidence supports their interpretation as intraspecific lineages rather than distinct species. The COI phylogeny grouped all lineages within a well-supported monophyletic clade (*pp* = 1), genetic distances among lineages (within *T. iheringi*) were lower than those typically observed among species, and no morphological differences were detected. In particular, species identification based on penial stylet morphology remained consistent across lineages, and PCoA revealed no clear morphological segregation. Moreover, all analyzed snails are recognized hosts of *T. iheringi,* and, except for RA, all sampled populations occur within the known distribution range of the species.

*Temnocephala iheringi* has been the subject of several morphological studies [10] (and references therein), and recent works have highlighted considerable morphological variability throughout its distribution range [11,12]. The multivariate analysis revealed partial structuring among specimens, with some tendency toward clustering according to both host species and geographic locality. However, substantial overlap among groups indicates that neither factor alone fully explains the observed morphometric variation. Because most localities were represented by a single host species, the effects of host association and geography cannot be clearly disentangled. Nevertheless, geographically distant populations tended to occupy distinct regions of the ordination space, suggesting that geographic isolation may contribute to morphological differentiation in the penial stylet.

During this study, two specimens from the Arapiuns River (Brazil) were found not to belong to *Temnocephala iheringi*, based on morphological characters and their genetic divergence, suggesting that they represent a different taxonomic entity. Unfortunately, the morphology of the penial stylet in these specimens does not allow a reliable species-level identification.

Interspecific genetic distances for the COI gene vary across different flatworm groups. These distances varied from 6.3 to 24.4% [39], 10.6 to 22% [40], and 12.5 to 15.7% [41] for triclads, and 9.4% [42] and 10.72 to 22.14% [43] for polyclads. Similar ranges have been observed in trematodes and cestodes (4.3 to 21.4%; [44]), while distances of approximately 7.7% have been found among digenean species within the same family [45]. The genetic distances found in this study for *Temnocephala* spp. (15.42–18.45%) fall toward the upper end of these known ranges. Interestingly, the distance between *Temnocephala* spp. and *Temnosewellia fax* (18.46–19.76%) was not significantly higher than that found between Neotropical species.

Previous studies have reported intraspecific distances of 10–12% for temnocephalans [46]. In the present study, *T. iheringi* showed a mean intraspecific distance of 6.38%, with values ranging from 0.13 to 12.69% among populations. Distances among lineages (4.7–12.5%) fell within the range of intraspecific distances.

*Temnocephala iheringi* exhibited high haplotype diversity (Hd: 0.92), with 31 haplotypes identified from 59 specimens across five different host species and 12 sample localities. This diversity is slightly higher than that reported for a related genus in the Australian region, where 17 haplotypes were found among 61 specimens (Hd: 0.89). Notably, the Australian sequences were derived from a single host species narrowly restricted to three mountaintops, *Euastacus robertsi* Monroe, 1977 [46].

All groups recovered in the haplotype network match the lineages identified by phylogenetic inference and species delimitation analysis. The Lineage L1, composed mainly of specimens from Buenos Aires Province, contained the highest number of haplotypes (15), with low differentiation among them, and no evident internal structure associated with either sampling localities or host species (*P. canaliculata* and *P. maculata*). All localities from this lineage have at least two haplotypes, except for Saavedra Park, where all sampled specimens shared a single haplotype, which was also present in four other localities within the group. This locality is noteworthy because it is an artificial pond with no direct connection to other water bodies. These results suggest a high degree of genetic homogeneity among populations from Buenos Aires province. This pattern is consistent with Avise’s category IV, defined as “shallow gene tree, lineages sympatric, a pattern that is expected for high-gene-flow species of modest or small effective size whose populations have not been sundered by long-term biogeographic barriers” [47]. However, L1 also included three sequences (unique haplotypes) from Entre Ríos Province (RN14).

Lineage L2 comprised the two remaining sequences from RN14 and a single sequence from LP (Buenos Aires Province). The latter is the only unique haplotype detected in that site, since the remaining specimens from LP share the dominant haplotype of L1. All specimens from RN14 showed unique haplotypes and were obtained from different host individuals (*P. canaliculata*). Because R14 is a temporary pond in an area subject to periodic flooding, it is possible that the dispersal of host snails among water bodies, potentially facilitated by hydrological connectivity through flood events and the Río de la Plata system, may promote gene flow between populations from Entre Ríos and Buenos Aires provinces. Since LP is not part of the Plata Basin, gene flow with the other localities could be related to instances of zoocoria or anthropic-mediated dispersion.

Lineages L4 (*P. canaliculata*) and L7 (*A. platae*) correspond to different populations, with four and three exclusive haplotypes each. This genetic separation matches the drainage basins to which these localities belong: LC to the Uruguay River Basin, and RV to the Paraná River Basin. L4 is well separated from L5 and L6, which are close geographically, but are associated with different hosts.

Lineage L5 comprises sequences from two geographically close sites: ESG and BLP, both with a single host species, *Pomacea megastoma*. It is composed of two haplotypes, one of which was shared by all specimens from BLP and by most of ESG. In contrast, ESG was also represented by the lineage L6, composed of a highly divergent, unique haplotype separated from L5 by numerous substitutions. Notably, this specimen was collected from the same host individual as two specimens with the dominant haplotype of L5.

Regarding the specimens from RA (Brazil), they formed a distinct and well-supported group (L3), clearly separated from all other lineages. This locality is also geographically distant from the remaining localities, and all specimens were collected from *Pomacea linnaei*, the host species represented exclusively at this locality. In addition, another unidentified species of *Temnocephala* was detected at the same locality.

Given that all delimited groups are connected through missing central nodes, it could be inferred that the most ancient haplotypes are currently rare or absent from the sampled populations [48]. A possible association by host species could be suggested, since the groups associated with *Pomacea megastoma* and *Asolene platae* are separated from those associated with *Pomacea canaliculata*. However, there is no separation between the latter and the specimens associated with *Pomacea maculata*. On the other hand, given that only one host species was sampled at each site, the information available is insufficient to clearly disentangle the relative effects of host association and geographic distribution on the observed genetic structure.

Due to the large geographic distances between sampling sites, we expected to find a higher correlation between genetic and geographic distances; however, no significant correlation was found.

The hosts, and with them their symbionts, disperse by other means besides direct communication between water bodies. For example, flooding is a well-known dispersal mechanism allowing snails to move from rivers and lagoons into adjacent areas, subsequently colonizing temporary ponds and other habitats. Ampullariid gastropods, particularly some *Pomacea* species, show strong resistance to adverse environmental conditions such as desiccation and low temperatures, and both *P. canaliculata* and *P. maculata* (and their symbionts) are highly invasive with high dispersion capacity [49,50,51].

On the other hand, the mechanisms underlying temnocephalans’ dispersal are not entirely well known. Exotic temnocephalans have been reported from several regions worldwide, likely introduced accidentally through human-mediated transport together with their hosts, both in aquaculture facilities and in established populations inhabiting natural water bodies [52]. They have also been recorded as associated with native hosts, different from those with which they were originally introduced [53,54,55]. Host-switching may be facilitated by both phylogenetic relatedness among hosts and geographic proximity [19], which could explain the pattern observed in the specimens from L1, associated with the species *P. canaliculata* and *P. maculata*, two species that show a significant biogeographic distribution overlap and that hybridize easily [13]. Also, temnocephalan eggs have been found in aquatic birds’ feces and, although their viability has not been tested [56], it is known that they are more resistant to adverse conditions than the adults [46]. Ložek et al. [57] recorded infestation of a new host species through temnocephalan eggs, as well as the jump to other hosts, kept separately but in the same water circulation system, pointing out the high dispersal potential of these symbionts under suitable conditions.

The high genetic diversity and shared haplotypes among populations evidenced in this study could indicate a considerable dispersal capacity in *Temnocephala iheringi*. Fišer et al. [58] suggested that extant temnocephalids can maintain gene flow among populations more effectively than their shrimp hosts. A similar pattern could potentially occur in temnocephalans associated with gastropods. However, while temnocephalans on shrimps generally inhabit the external body surface, those associated with snails live mainly within the mantle cavity, leaving the host only to deposit eggs on the shell. Nonetheless, ampullariid snails tend to exhibit gregarious behavior and prolonged copulation, conditions that could facilitate worm transmission among hosts.

## 5. Conclusions

This study provides the first molecular characterization of Neotropical temnocephalans at the population level, confirming that the COI mitochondrial marker is a robust tool for identifying species within the Temnocephalidae family. Our findings reveal that *Temnocephala iheringi* possesses high haplotype diversity and partial association with host species and geography. While some populations exhibit clear lineage separation tied to specific drainage basins and host species (such as *Pomacea megastoma* and *Asolene platae*), the low mitochondrial differentiation observed in lineages associated with *P. canaliculata* and *P. maculata* underscores the significant role of host mobility in shaping symbiont evolutionary dynamics. However, further study is needed, involving wider geographic coverage, overlapping host species, and additional molecular markers to clearly elucidate the relative effects of host association and geographic distribution on the genetic differentiation of *T. iheringi*. Lastly, despite the high genetic variability, the relative stability of the penial stylet morphology across these lineages suggests that traditional diagnostic features remain reliable.

## Figures and Tables

**Table 1:** Sampling sites, host species of *Temnocephala iheringi*, and the number of host specimens sampled.

Province/State, Country	Site Code	Locality	Latitude	Longitude	Sampling Date	Host Species (n° of Collected Specimens)
Buenos Aires, Argentina	AZ	Zapata Stream and Provincial Route 11	−34.98844	−57.71675	12 January 2017	*Pomacea canaliculata* (14)
	AT	Atalaya	−35.01567	−57.52769	16 July 2013	*Pomacea canaliculata*
	LB	La Balandra (stream)	−34.92964	−57.71329	8 November 2018	*Pomacea canaliculata* (7)
	RNO	National Park Ciervo de los Pantanos	−34.23739	−58.88055	30 April 2015	*Pomacea canaliculata*
	PS	Saavedra Park, La Plata	−34.9305	−57.94187	4 April 2017	*Pomacea canaliculata* (10)
	RP	Río de la Plata, Regatas Club, Ensenada	−34.84063	−57.92176	25 April 2017	*Pomacea maculata* (11)
	LP	Laguna de los Padres	−37.93333	−57.73333	January 2022	*Pomacea canaliculata*
Entre Ríos, Argentina	BLP	Las Palmeritas Beach	−31.23887	−57.95624	24 May 2019	*Pomacea megastoma* (2)
	ESG	Salto Grande Reservoir	−31.2373	−57.95278	9 February 2017	*Pomacea megastoma* (3)
	RN14	National Route 14, Km 19	−33.35344	−58.72763	10 September 2016	*Pomacea canaliculata* (14)
	LC	Provincial Route 4 towards Los Charrúas, Concordia	−31.33638	−58.104	29 March 2014	*Pomacea canaliculata*
	RV	Riacho Victoria, Quinto Cuartel neighborhood, Victoria	−32.64479	−60.1554	11 February 2020	*Asolene platae* (5)
Pará, Brazil	RA	Arapiuns River; near Vila Franca	−2.33373	−55.03345	20 November 2013	*Pomacea linnaei* (3)

**Table 2:** Collection and sequence data for *Temnocephala iheringi* specimens examined in this study. Number of sequenced specimens (T), number of hosts from which the sequenced temnocephalids were extracted (H), mean abundance (total number of *T. iheringi* divided by the total number of hosts examined), GeneBank accession numbers (* only forward strand sequenced), and MLP catalog numbers. (?): data not available.

Province/State, Country	Locality	Host	*Temnocephala* Species (Mean Abundance)	T, H	GenBank Accession Numbers/(MLP Catalog Number)
Buenos Aires, Argentina	CB	*P. argentinus*	*T. digitata*		PZ543717 (MLP He 7217); PZ543754; PZ543755
	■ AZ	*P. canaliculata*	*T. iheringi* (5.50)	T: 5; H: 5	PZ543720; PZ543721; PZ543722; PZ543723; PZ543724 (MLP-He 8375)
	■ AT	*P. canaliculata*	*T. iheringi* (?)	T: 1; H: 1	PZ543746 (MLP-He 8374)
	■ LB	*P. canaliculata*	*T. iheringi* (3.14)	T: 6; H: 4	PZ543718; PZ543725; PZ543726; PZ543739; PZ543740; PZ543741 (MLP-He 8376)
	■ RNO	*P. canaliculata*	*T. iheringi* (?)	T: 4; H: ?	PZ543745; PZ543751; PZ543752; PZ543753 (MLP-He 8377)
	■ PS	*P. canaliculata*	*T. iheringi* (13.60)	T: 5; H: 2	PZ543780 *; PZ543777 *; PZ543776 *; PZ543779 *; PZ543778 * (MLP-He 8382)
	■ RP	*P. maculata*	*T. iheringi* (8.00)	T: 7; H: 3	PZ543731; PZ543732; PZ543733; PZ543774 *; PZ543772 *; PZ543775 *; PZ543773 * (MLP-He 8378)
	■ LP	*P. canaliculata*	*T. iheringi* (?)	T: 5; H: ?	PZ543766; PZ543767; PZ543768; PZ543769; PZ543770 (MLP-He 8379)
Entre Ríos, Argentina	■ BLP	*P. megastoma*	*T. iheringi* (34.00)	T: 3; H: 2	PZ543742; PZ543743; PZ543744 (MLP-He 8384)
	■ ESG	*P. megastoma*	*T. iheringi* (7.00)	T: 6; H: 2	PZ543719; PZ543734; PZ543735; PZ543736; PZ543737; PZ543738 (MLP-He 8385)
	■ RV	*A. platae*	*T. iheringi* (0.60)	T: 3; H: 2	PZ543756; PZ543757; PZ543758 (MLP-He 8386)
	■ RN14	*P. canaliculata*	*T. iheringi* (9.36)	T: 5; H: 5	PZ543716; PZ543727; PZ543728; PZ543729; PZ543730 (MLP-He 8387)
	■ LC	*P. canaliculata*	*T. iheringi* (?)	T: 4; H: ?	PZ543747; PZ543748; PZ543749; PZ543750 (MLP-He 8388)
Pará, Brazil	■ RA	*P. linnaei*	*T. iheringi* (?)	T: 7; H: 3	PZ543759; PZ543760; PZ543761; PZ543764; PZ543765 (MLP-He 8389)
			*Temnocephala* sp.		PZ543762; PZ543763 (MLP-He 8390)

**Table 3:** Number of haplotypes of *T. iheringi* per locality.

Locality	Number of Sequences	Number of Haplotypes	Number of Unique Haplotypes
AZ	5	3	2 (PZ543723, PZ543724)
AT	1	1	-
LB	6	4	1 (PZ543718)
RNO	4	3	2 (PZ543751, PZ543753)
PS	5	1	-
RP	7	6	3 (PZ543732, PZ543774, PZ543773)
LP	5	2	1 (PZ543768)
BLP	3	1	-
ESG	6	3	2 (PZ543735, PZ543737)
RV	3	3	3
RN14	5	5	5
LC	4	4	4
RA	5	3	1 (PZ543761)

## Data Availability

The datasets generated and analyzed during the current study are included in the article/Supplementary Materials. Newly generated sequences are available on GenBank. Further inquiries can be directed to the corresponding authors.

## Supplementary Materials

The following supporting information can be downloaded at: https://www.mdpi.com/article/10.3390/biology15131020/s1, Figure S1: Microphotographs of the penial stylet of *Temnocephala iheringi*. a, PZ543764. b, PZ543765. c, PZ543720. d, PZ543721. e, PZ543722. f, PZ543723. g, PZ543725. h, PZ543730. i, PZ543727. j, PZ543726. k, PZ543729. l, PZ543728. m, PZ543732. n, PZ543733. o, PZ543734. Lineages: a, b: L3; c, d, e, f, g, h, i, j, l, m, n: L1; k: L2; o: L5. Scale bar 50 μm; p, PZ543737. q, PZ543740. r, PZ543742. s, PZ543744. t, PZ543747. u, PZ543750. v, PZ543753. w, PZ543758. x, PZ543743. y, PZ543766. z, PZ543767. aa, PZ543768. bb, PZ543770. Lineages: aa: L2; p: L6; bb, q, v, y, z: L1; r, s, x: L5; t, u: L4; w: L7. Scale bar 50 μm; Figure S2: Maximum likelihood COI gene tree for *Temnocephala iheringi*. Branch support values are Sh-aLRT/Ultrafast Bootstrap; Table S1: Genetic alignment; Table S2: Genetic distances; Table S3: Geographic distances between sampling localities.

## Appendix A

**Table A1:** Penial stylet measurements (in µm) from *T. iheringi* vouchers used in principal coordinates analysis.

Lineage	Specimen	Stylet Total Length	Introvert Length	Shaft Width at Base	Introvert Width at Base	Introvert Maximum Width
L1	PZ543720	175	57.5	-	32.5	47.5
L1	PZ543721	-	52.5	-	30	42.5
L1	PZ543722	192.5	55	157.5	27.5	42.5
L1	PZ543723	220	57.5	142.5	30	45
L1	PZ543724	170	52.5	150	27.5	50
L1	PZ543725	197.5	47.5	150	25	40
L1	PZ543726	215	57.5	140	27.5	37.5
L1	PZ543727	192.5	52.5	145	25	42.5
L1	PZ543728	190	60	142.5	32.5	47.5
L2	PZ543729	207.5	55	150	30	42.5
L1	PZ543730	222.5	62.5	182.5	30	40
L1	PZ543731	212.5	60	162.5	30	35
L1	PZ543732	170	52.5	152.5	27.5	40
L1	PZ543733	205	55	162.5	30	37.5
L5	PZ543734	150	50	117.5	25	40
L6	PZ543737	180	55	132.5	27.5	42.5
L1	PZ543740	197.5	52.5	140	25	37.5
L5	PZ543742	167.5	47.5	127.5	25	40
L5	PZ543743	165	47.5	130	25	42.5
L5	PZ543744	172.5	47.5	122.5	25	42.5
L4	PZ543747	162.5	50	137.5	35	52.5
L4	PZ543748	-	50	-	22.5	50
L4	PZ543749	-	47.5	-	25	45
L4	PZ543750	187.5	52.5	142.5	32.5	45
L1	PZ543753	-	57.5	117.5	32.5	47.5
L7	PZ543758	152.5	47.5	120	22.5	35
L3	PZ543764	225	62.5	100	27.5	47.5
L3	PZ543765	200	50	127.5	30	50
L1	PZ543767	197.5	62.5	150	30	47.5
L2	PZ543768	207.5	55	172.5	27.5	45
L1	PZ543766	202.5	55	140	32.5	50
L1	PZ543770	212.5	50	150	22.5	45
