# Supplementary figures and images for: Unraveling the Population Structure of Temnocephala iheringi Across Host Associations and Geographic Regions
Source: Biology (Basel). 2026 Jun 26;15(13):1020. doi: 10.3390/biology15131020 (PMC13360199; doi:10.3390/biology15131020)

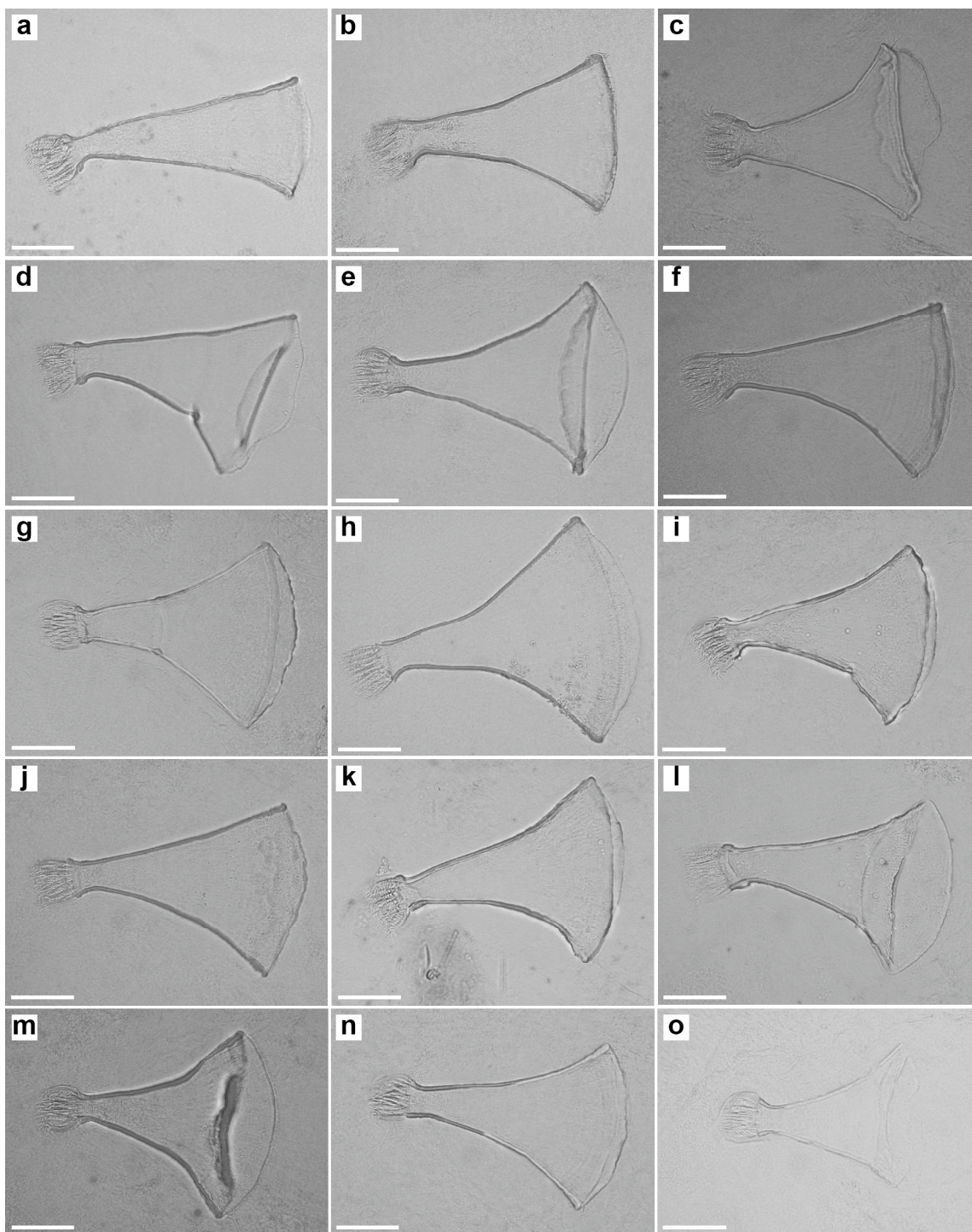

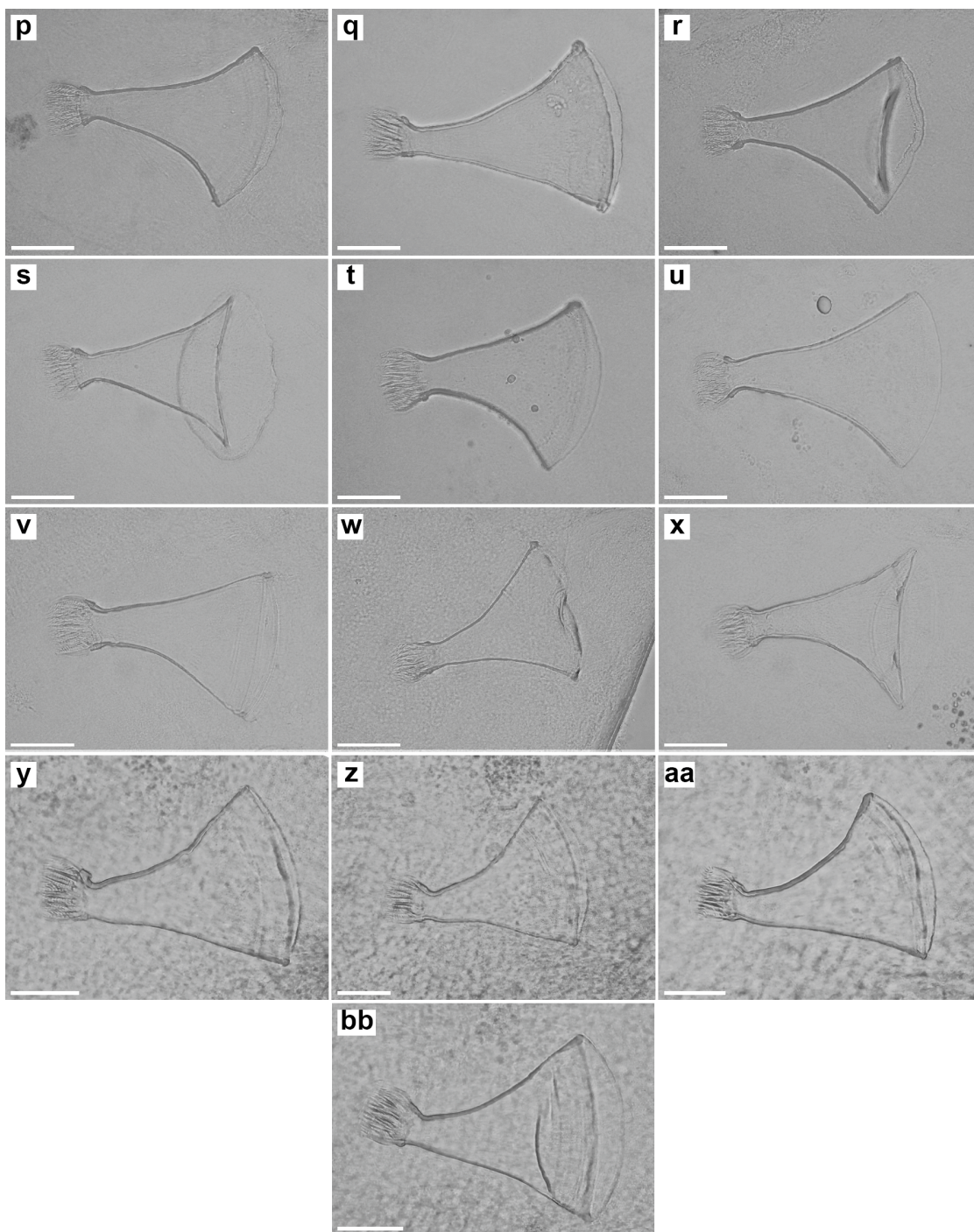

Supplement: Supplementary file 1 [file biology-15-01020-s001.zip › Figure S1.pdf]

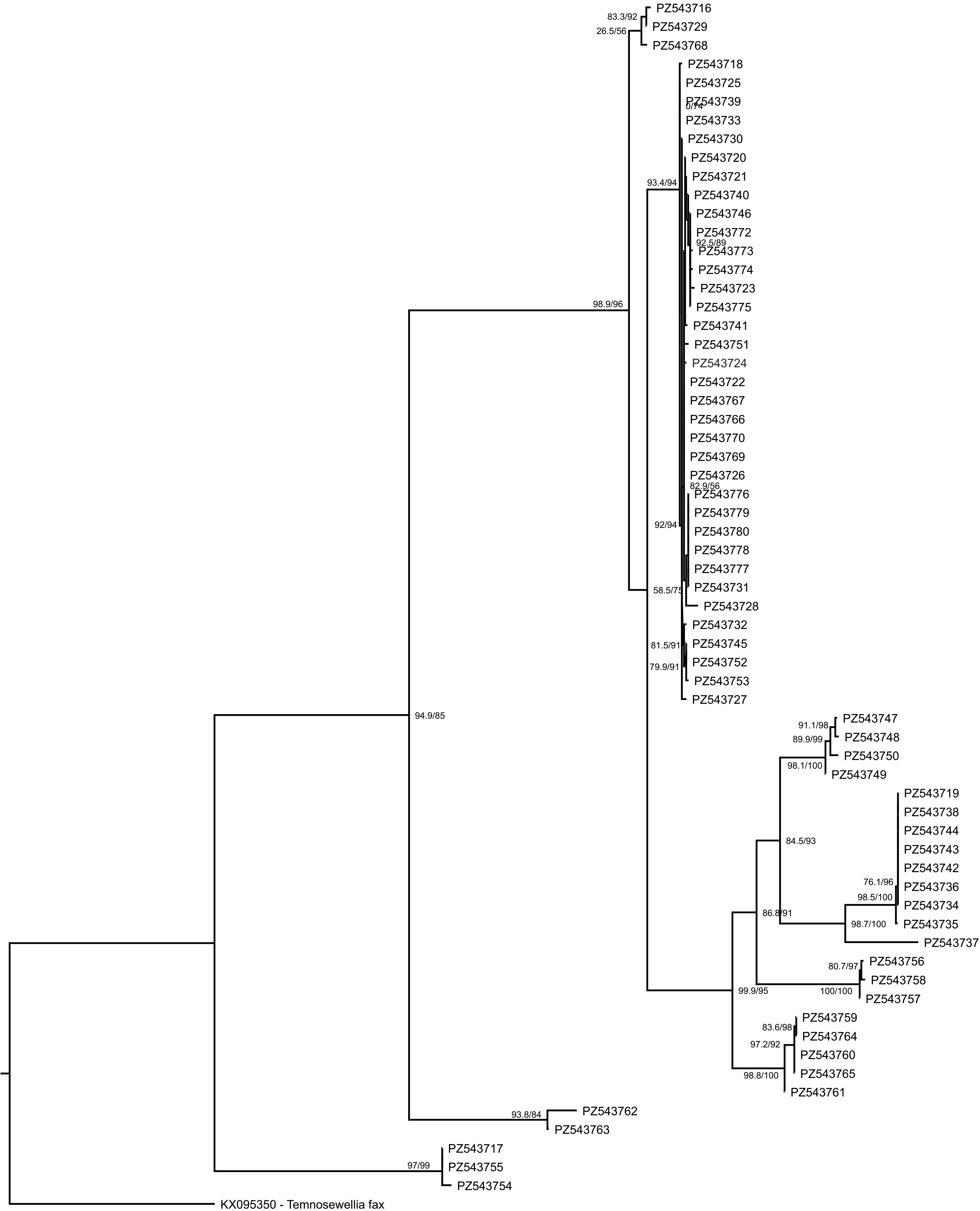

Supplement: Supplementary file 1 [file biology-15-01020-s001.zip › Figure S2.pdf]
